# Effects of Influenza Vaccine on the Immune Responses to SARS-CoV-2 Vaccination

**DOI:** 10.3390/vaccines12040425

**Published:** 2024-04-17

**Authors:** A. Riccomi, C. M. Trombetta, M. Dorrucci, D. Di Placido, N. Sanarico, F. Farchi, R. Giuseppetti, U. Villano, C. Marcantonio, S. Marchi, A. Ciaramella, P. Pezzotti, E. Montomoli, C. Valdarchi, A. R. Ciccaglione, S. Vendetti

**Affiliations:** 1 Department of Infectious Diseases, Istituto Superiore di Sanità, 00161 Rome, Italymaria.dorrucci@iss.it (M.D.); daniela.diplacido@guest.iss.it (D.D.P.); francesca.farchi@iss.it (F.F.); umbertina.villano@iss.it (U.V.);; 2 Department of Molecular and Development Medicine, University of Siena, 53100 Siena, Italyserena.marchi2@unisi.it (S.M.);; 3 VisMederi Research Srl, 53100 Siena, Italy; 4 Center for Control and Evaluation of Medicines, Istituto Superiore di Sanità, 00161 Rome, Italy; nunzia.sanarico@iss.it; 5 Research Coordination and Support Service, Istituto Superiore di Sanità, 00161 Rome, Italy; antonio.ciaramella@iss.it; 6 VisMederi Srl, 53100 Siena, Italy

**Keywords:** influenza vaccine, SARS-CoV-2 vaccine, immune response to vaccination

## Abstract

A number of studies have suggested that influenza vaccination can provide protection against COVID-19, but the underlying mechanisms that could explain this association are still unclear. In this study, the effect of the 2021/2022 seasonal influenza vaccination on the immune response to the booster dose of anti-SARS-CoV-2 vaccination was evaluated in a cohort of healthy individuals. A total of 113 participants were enrolled, 74 of whom had no prior COVID-19 diagnosis or significant comorbidities were considered for the analysis. Participants received the anti-influenza tetravalent vaccine and the booster dose of the anti-SARS-CoV-2 vaccine or the anti-SARS-CoV-2 vaccine alone. Blood was collected before and 4 weeks after each vaccination and 12 weeks after SARS-CoV-2 vaccination and analyzed for anti-flu and anti-spike-specific antibody titers and for in vitro influenza and SARS-CoV-2 neutralization capacity. Results indicated an increased reactivity in subjects who received both influenza and SARS-CoV-2 vaccinations compared to those who received only the SARS-CoV-2 vaccine, with sustained anti-spike antibody titers up to 12 weeks post-vaccination. Immune response to the influenza vaccine was evaluated, and individuals were stratified as high or low responders. High responders showed increased antibody titers against the SARS-CoV-2 vaccine both after 4 and 12 weeks post-vaccination. Conversely, individuals classified as low responders were less responsive to the SARS-CoV-2 vaccine. These data indicate that both external stimuli, such as influenza vaccination, and the host’s intrinsic ability to respond to stimuli play a role in the response to the vaccine.

## 1. Introduction

Vaccines are considered one of the most effective life-saving medical interventions in medical history [1], playing a crucial role in preventing infections and diseases. However, the variability in vaccine efficacy among individuals underscores the importance of understanding more deeply the mechanisms that regulate immune responses to vaccination. Predicting who will respond optimally to a given vaccine is a critical aspect of vaccine development. The potential interaction between vaccines and infectious agents has lately attracted a lot of attention. A growing number of studies indicate that some vaccines possess significant non-specific protective effects, offering broader immunity beyond their primary targets. Vaccines such as Bacillus Calmette–Guérin (BCG) [2], measles vaccines, and the oral polio vaccine have been demonstrated to have protective effects against unrelated infectious diseases [3,4], highlighting the potential of using existing vaccines to provide protection against a range of pathogens.

In line with this concept, recent studies suggested a potential link between influenza vaccination and decreased COVID-19 incidence and severity [5,6,7]. This suggests that influenza vaccination may potentially convey partial protection against COVID-19. A systematic review and meta-analysis of observational studies reveals an inverse association between influenza vaccination and COVID-19 risk, and it analyses the association with clinical outcomes such as mortality, hospitalization, and admissions in intensive care of SARS-CoV-2 infected subjects. However, more evidence-based studies are needed to investigate the underlying mechanisms that could explain this association [8].

Despite being the most effective measure for preventing influenza and its complications, influenza vaccination coverage remains relatively low, particularly among fragile individuals [9]. The response to influenza virus vaccination exhibits significant variability within populations, leaving certain individuals inadequately protected even when the seasonal vaccine aligns with the prevalent influenza viral strains [10].

The increase in antibody titers against influenza antigens after the vaccination and the achievement of an antibody titer of 40 are generally regarded as a protective threshold level, beyond which there is a 50% or greater reduction in the possibility of contracting influenza infection. This is related to intrinsic factors such as age [11,12], gender [13], pre-existing antibody titers [11,12], and vaccination history [14,15]. However, most of the variability in antibody responses to influenza virus vaccination remains unexplained [12]. Identifying individuals with high or low responsiveness to immunization and understanding the extent of this responsiveness holds the potential for optimizing existing vaccination strategies.

In this study, we explored the association between influenza vaccination and the immune responses to the booster dose of the anti-SARS-CoV-2 vaccine in a cohort of healthy individuals, exploring the hypothesis that the ability to respond to influenza vaccination has an impact on the capacity to respond to the anti-SARS-CoV-2 vaccination.

## 2. Materials and Methods

### 2.1. Cohort Description and Sampling

A study cohort of healthy individuals was recruited (*N* = 113) between October 2021 and January 2022 by their general practitioners (GPs) located in the area of Rome municipality, Lazio region, Italy. Study participants received the booster dose of the SARS-CoV-2 mRNA vaccine and 54 of them were also vaccinated with the quadrivalent inactivated influenza vaccine (QIV) Flucelvax (Seqirus), which contained neuraminidase and hemagglutinin from the following viral strains: A/Wisconsin/588/2019, A/Cambodia/e0826360/2020, B/Phuket/3073/2013, and B/Washington/02/2019 [16]. Study participants diagnosed with cancer, immunological disorders, receiving immunosuppressive therapy, subjected to transplant or vaccinations in the last 6 months, suffering from chronic disease or viral or bacterial infections, and pregnant women were excluded from this study. Influenza vaccine was injected before the booster dose of the SARS-CoV-2 vaccine, and the participants were bled before and after 28 days after receiving the anti-flu vaccination. Blood samples were collected in all participants before (T0), one (T1) month, and three (T3) months after receiving the booster dose of SARS-CoV-2 vaccination. After each vaccination, we tracked their humoral immune responses against the influenza antigens included in the vaccine and against the SARS-CoV-2 virus.

### 2.2. Ethics Statement

This study was approved by the ethics committee of the Istituto Superiore di Sanità, A00ISS-15/03/2021-0010084. This study was conducted in accordance with applicable laws and regulations, including, without limitation, the International Conference on Harmonisation (ICH) Guideline for Good Clinical Practice (GCP) and the ethical principles derived from the Declaration of Helsinki. At the time of enrolment, patients were required to sign an informed consent that included the acceptance of sampling procedures and the collection and management of data for epidemiologic and scientific purposes. Patient data were anonymized.

### 2.3. Serum Samples

Anonymized human serum samples (*N* = 113) were collected and stored in compliance with Italian ethics law. Positive controls were provided by the National Institute for Biological Standards (NIBSC) in the form of influenza sheep hyperimmune serum samples: A/Wisconsin/588/2019 (H1N1, 09/152) (A/Wis); A/Cambodia/e0826360/2020 (H3N2, 13/110) (A/Cam); B/Phuket/3073/2013 (B/Yamagata lineage, 11/136) (B/Phu); and B/Washington/02/2019 (B/Victoria lineage, 19/218). Negative controls consisted of human serum without IgA, IgM, and IgG (Sigma-Aldrich, Milan, Italy).

### 2.4. Hemagglutination Inhibition Assay

All serum samples, including the sheep hyperimmune serum samples and negative control, underwent pre-treatment with receptor-destroying enzyme (RDE) (at a ratio of 1:5) obtained from Vibrio cholerae (C8772, Sigma-Aldrich, Milan, Italy) for 18 h at 37 °C in a water bath. Subsequently, they were heat-inactivated for 1 h at 56 °C in a water bath containing 8% sodium citrate (at a ratio of 1:4). Fresh turkey red blood cells were centrifuged twice, washed with a saline solution (0.9%), and adjusted to a final dilution of 0.35%. Serum samples were initially diluted 1:10 and then subjected to 2-fold diluted in duplicate with saline solution (0.9%) in a 96-well plate; each well received 25 μL of standardized viral antigen (4 HA units/25 μL), followed by incubation at room temperature for 1 h. Red blood cells were then added, and after 1 h of incubation at room temperature, the plates were evaluated for the inhibition of agglutination. The antibody titer was expressed as the reciprocal highest serum dilution that showed complete inhibition of agglutination. Given that the starting dilution was 1:10, the lower limit of the detectable antibody titer was 10. Results below this threshold were conventionally expressed as 5 for calculation purposes, corresponding to half the lowest detection threshold.

### 2.5. Influenza Virus Microneutralization Assay

Madin–Darby canine kidney (MDCK) cells were cultured for a maximum of 30 passages in Eagle’s minimal essential medium (EMEM) supplemented with 10% fetal bovine serum (FBS), 2 mmol/L L-glutamine, 1% non-essential amino acid solution, and 100 U/mL penicillin–streptomycin. The MDCK cell cultures were maintained at 37 °C in 5% CO_2_. Serum samples, previously heat-inactivated at 56 °C for 30 min, were subjected to 2-fold dilution with EMEM culture medium supplemented with 0.5% FBS in a 96-well plate. Each sample was mixed with an equal volume of virus (100 TCID50/well) and incubated for 1 h at 37 °C in 5% CO_2_. Subsequently, MDCK cell suspension (1.5 × 10^5^ cells/mL) was added to the plates, which were then incubated at 37 °C, 5% CO_2_ for 5 days. After incubation, the plates were examined by optical microscopy to assess cytopathic effect (CPE). A CPE exceeding 50% indicated infection. The titer was expressed as the reciprocal of the last dilution that showed inhibition of cytopathic effect.

### 2.6. SARS-CoV-2 IgG Immunoassay Testing

Serum samples underwent testing for the presence of SARS-CoV-2 IgG antibodies targeting the receptor binding domain (RBD) of the spike (S) protein and the nucleocapsid (NP) protein using the Abbott SARS-CoV-2 IgG assay (Abbott, Park, IL, USA) on the Abbott Architect i2000SR automated analyzer according to the manufacturer’s instructions. Results were interpreted as positive when higher than or equal to the cut-off index value of 50 AU for the anti-S and 1.4 AU for the anti-NP IgG antibody titer.

### 2.7. SARS-CoV-2 Virus Neutralization Assay

The virus neutralization (VN) assay was conducted following previously reported methods [17]. Briefly, serum samples were heat-inactivated at 56 °C for 30 min and then subjected to 1:10 dilution. Subsequently, the diluted samples were mixed with an equal volume of SARS-CoV-2 (2019-nCov/Italy-INMI1 strain) viral solution containing 100 Tissue Culture Infectious Dose 50% (TCID50). After 1 h of incubation at room temperature, 100 μL of virus–serum mixture was added to a 96-well plate containing VERO E6 cells with 80% confluency. The plates were then incubated for 3 days at 37 °C, with 5% CO_2_ in a humidified atmosphere. After incubation, the plates were examined for the presence or absence of cytopathic effect (CPE) using an inverted optical microscope. A CPE exceeding 50% indicated infection. The VN titer was expressed as the reciprocal of the highest serum dilution that showed protection from viral infection and CPE.

### 2.8. Statistical Analysis

We summarized categorical variables by frequency and percent, while continuous variables by median and interquartile ranges (IQRs). We performed the description of the study population by main study groups, i.e., individuals who received both vaccines (flu vaccine and SARS-CoV-2 vaccine) vs. those who received only SARS-CoV-2 vaccine. Double-vaccinated individuals were stratified by considering the median of the HI titer of each specific antigen 28 days post-vaccination (D28) as a reference. The median of the HI titer is different for each antigen, and this might be due to the different immunogenicity of the viral antigens, the vaccination history of the participants, and the virus circulation in the previous years. The study population was stratified into five groups: individuals with no specific HI titer above the specific median at D28 (0, n = 12) and individuals with respectively, one (1, n = 13), two (2, n = 9), three (3, n = 16), and four (4, n = 4) specific HI titers above the specific median. Participants belonging to groups 0 or 1 were classified as low responders (LRs), and participants belonging to groups 2, 3, or 4 were classified as high responders (HRs). We assessed differences between two groups by non-parametric Mann–Whitney U test. We applied Kruskal–Wallis test when the number of the groups was higher than two; we also applied Bonferroni adjustment when appropriate. In order to investigate the relationship between the response to the S protein and to the four flu-antigens, we applied a multiple-regression model after one (T1) and three (T3) months post-SARS-CoV-2 vaccination. In detail, we considered the natural logarithm ln of anti-S titer as the response variable, while covariates included the ln of anti-flu for all four antigens, as well as their interactions with each antigen. Further, other interactions were considered in the model when resulting statistically significant, with one of the possible covariates: anti-spike at baseline (≤its median vs. >its median), age (≤60 vs. >60), and sex (females vs. males). We used SAS/STAT version 9.4 (SAS Institute, Cary, NC, USA) for the statistical analyses.

## 3. Results

### 3.1. Characteristics of the Study Population

To evaluate the effects of the 2021/2022 seasonal influenza vaccine on the immune response to SARS-CoV-2 vaccination, we recruited participants from a cohort study of healthy individuals (*N* = 113) who did or did not receive QIV in autumn 2021 and the booster dose of the SARS-CoV-2 vaccine. Healthy participants without previous COVID-19 diagnosis and without significant comorbidities were considered for the analysis (*N* = 74) (Table 1). In particular, study participants diagnosed with cancer, immunological disorders, receiving immunosuppressive therapy, subjected to transplant or vaccinations in the last 6 months, suffering from chronic disease or viral or bacterial infections, and pregnant women were excluded from this study. Among the 74 participants, 50 (68%) were females and 24 (32%) were males, with a similar proportion between the two sexes in participants who received two vaccines (67% vs. 33%) and the ones who received one vaccine (70% vs. 30%) (Table 1); overall, median age was 54 years (range: 31–72 years; interquartile range: 49–60 years) with similar distribution among individuals who received two (median: 54, range: 49–60; interquartile 35–70) or one (median: 51, range: 49–56; interquartile 31–72) vaccine. Most participants have higher (72%) or middle/lower (28%) education, with a higher proportion of higher education among participants who received two vaccines as compared with the ones who received only one vaccine (76% vs. 60%). Regarding frailty, the majority (69%) of study participants declared to be fit at the time of enrolment, part of them declared to be very fit (20%), and a minority (11%) declared to manage well, with no substantial differences between participants who received two vaccines or one vaccine (Table 1). Regarding BMI, 57% had a normal weight, 35% were overweight, and 7% were obese. However, no significant differences in the proportion of overweight/obese participants were observed between participants who received two vaccines or one vaccine (Table 1). Smoking at the time of vaccination was declared by 16% of participants, with a higher number of smokers among participants who received only one vaccine (25% vs. 13%). The majority of study participants (62.3%) did not have respiratory symptoms during the current season. The most frequent diseases found in the study population were chronic cardiac diseases (28%), asthma (21%), thyroiditis (15%), hemoglobinopathy (9%), essential thrombocythemia, diabetes or anemia (6%), and renal diseases or coagulopathy (3%). Part of the participants enrolled in this study received the seasonal influenza vaccine before the booster dose of the SARS-CoV-2 vaccine, and they were bled before and after 28 days after receiving the flu vaccination. Blood samples were collected in all participants before (T0), one (T1), and three (T3) months after receiving the boosting dose of SARS-CoV-2 vaccination. After each vaccination, we tracked their humoral immune responses against the influenza antigens included in the vaccine and against the SARS-CoV-2 virus.

### 3.2. Subjects Who Received Influenza and COVID-19 Vaccinations Are More Responsive to the SARS-CoV-2 Vaccine

**Figure 1:**
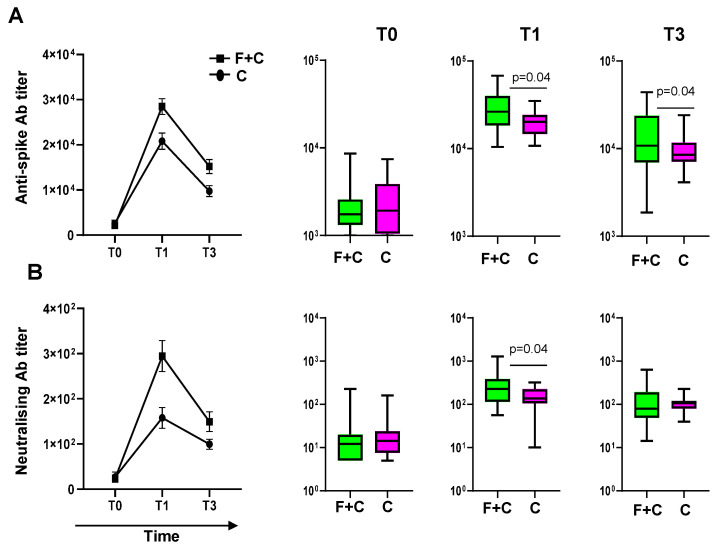
Effect of influenza vaccination on the response to the booster dose of anti-SARS-CoV-2 vaccine. Anti-spike antibody titers (**A**) and neutralizing antibody titers (**B**) in individuals who received both anti-flu and anti-SARS-CoV-2 vaccines (F+C) and those who only received the anti-SARS-CoV-2 vaccine (C) before receiving the mRNA-based SARS-CoV-2 vaccine (T0), after one month (T1) and after 3 months post-vaccination. Statistically significant differences between groups are shown with their relative *p*-values.

All participants who received two vaccines received the 2021/2022 seasonal anti-influenza tetravalent vaccine before the mRNA-based anti-SARS-CoV-2 vaccine. The time between the two vaccinations ranged between 1 and 70 days. Analysis of this interval did not reveal any significant time gap between the two vaccinations that could influence the levels of the response. Blood was collected by venous puncture before and 4 weeks after each vaccination and 12 weeks after SARS-CoV-2 vaccination. Subjects who received both influenza and COVID-19 vaccinations were more responsive to the SARS-CoV-2 vaccine after 4 weeks from the injection of the SARS-CoV-2 vaccine (T1) compared with individuals who received only the SARS-CoV-2 vaccine (mean anti-spike antibody titer: 28,871 ± 1885 vs. 20,823 ± 1804, respectively, *p* = 0.04) (Figure 1). The responses were evaluated in terms of anti-S-specific antibody titers by an immunometric assay (Figure 1A). Furthermore, an in vitro neutralization assay using authentic SARS-CoV-2 virus revealed higher titers of functional antibodies towards SARS-CoV-2 infection in individuals who received two vaccines (mean neutralizing antibody titer after 4 weeks: 301 ± 37 vs. 158 ± 23, respectively, *p* = 0.04) (Figure 1B). To assess whether an asymptomatic COVID-19 infection occurred before or during the trial, the anti-NP antibody titers were evaluated at each time point considered. Participants infected with SARS-CoV-2 showing an anti-NP antibody titer higher than 1.4 according to the assay used were excluded from the analysis (23% of the enrolled participants). The anti-S antibody titers decreased after the SARS-CoV-2 vaccine injection in all participants in accordance with the waning of the antibody titers observed after vaccination [18]. However, after three months (T3) from the injection, the antibody titer was higher (mean anti-spike antibody titer: 15,421 ± 1670 vs. 9745 ± 1212, respectively, *p* = 0.04) in the individuals who received both influenza and COVID-19 vaccines, suggesting that influenza vaccination also affects the durability of the immune response to the SARS-CoV-2 vaccine.

### 3.3. Quantitative and Qualitative Analysis of the Immune Responses to Seasonal 2021/2022 Influenza Vaccine

**Figure 2:**
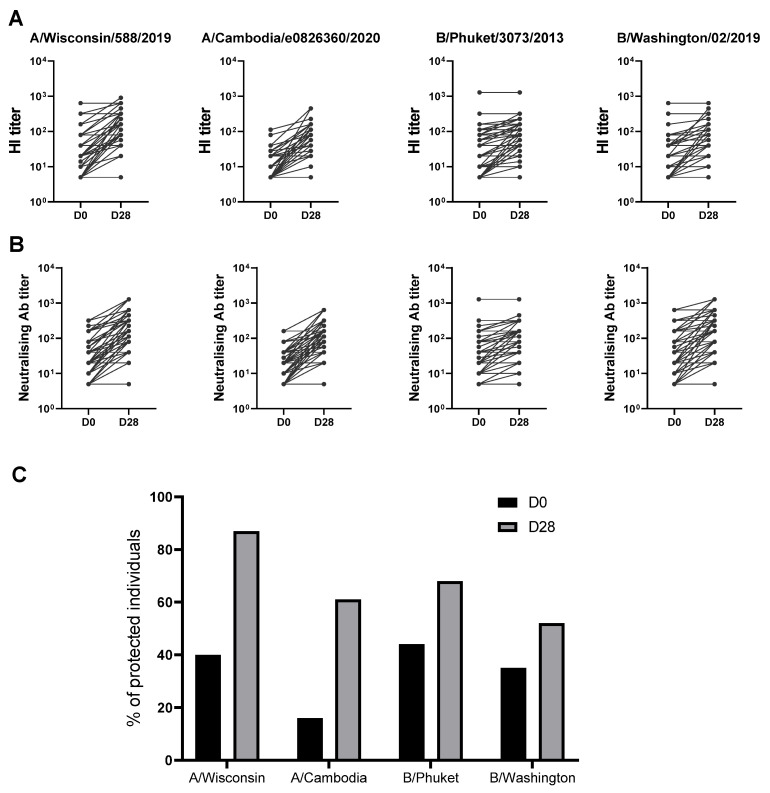
Humoral immunity to influenza vaccination. (**A**) Graph showing the basal (D0) HI titers and the response at day 28 (D28) for each one of the four influenza virus strains after vaccination. (**B**) Results from the microneutralization assays performed on the same individuals shown in A. The neutralization capacity is expressed as the reciprocal of the highest dilution of the donor’s serum at which virus infection is blocked. (**C**) Percentages of seroprotection in vaccinees at baseline (D0) and day 28 (D28) for each one of the four influenza virus strains.

Healthy participants received the 2021/2022 seasonal quadrivalent influenza vaccine following the Italian public health authority’s recommendation [16,19]. To evaluate the response to the vaccine, blood was collected at baseline before receiving the injection (D0) and 28 days after (D28). The hemagglutination-inhibition (HI) antibody titer at baseline (D0) and at D28 post-vaccination was evaluated (Figure 2A). The participants responded to vaccination with a significant increase in HI titers against all four antigens included in the vaccines tested at day 28 (Figure 2A). Following the current international guidelines for seroconversion (>4-fold increase in HI titers over baseline) and seroprotection (HI titers ≥ 40) [19,20], a high number of participants was already seroprotected at the baseline against the four antigens included in the vaccine. Differences between the baseline percentage of protection were observed for the different viral strains, with a lower percentage of protection found for the A/Cambodia/H3N2 strain (20%). Overall, the percentage of protection increases for all strains included in the vaccine, even if at different extensions after 28 days post-vaccination (Figure 2C). The high baseline titers can result from previous exposure to the influenza virus or earlier vaccinations. Furthermore, the HI results were supported by the microneutralization assays (Figure 2B), showing that the influenza-specific antibodies produced following vaccination are functional and have virus-neutralization capacity.

**Figure 3:**
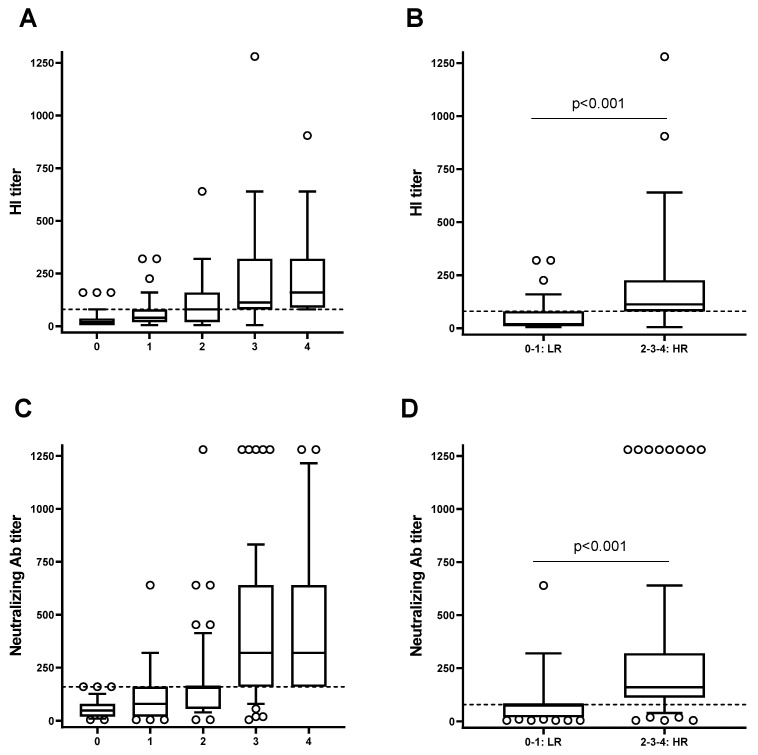
Stratification of the subjects based on the response to the four influenza virus antigens. The study population was stratified into five groups considering as a reference the median of HI titer (**A**,**B**) and the microneutralization titer (MN) (**C**,**D**) of each post-vaccination specific viral antigen: individuals with no specific HI or MN titer above the specific median post-vaccination (D28) (0) and individuals with respectively, one (1), two (2), three (3), and four (4) specific HI or MN titers above the specific median at D28 post-vaccination. Participants belonging to groups 0 or 1 were classified as low responders (LRs), and participants belonging to groups 2, 3, or 4 were classified as high responders (HRs). Dashed lines are means of medians of the four antigens and circles are outliers. The differences between LRs and HRs are statistically significant (*p* < 0.001).

Due to the elevated variability in the response to the influenza vaccine among individuals, double-vaccinated individuals were stratified according to their capacity to respond to the flu vaccination by considering as a reference the median of HI titer of each post-vaccination specific antigen. The study population was stratified into five groups: individuals with no specific HI titer above the specific median post-vaccination (D28) (0, n = 12) and individuals with respectively one (1, n = 13), two (2, n = 9), three (3, n = 16) and four (4, n = 4) specific HI titers above the specific median at D28 post-vaccination (Figure 3A). Participants belonging to groups 0 or 1 were classified as low responders (LRs), and participants belonging to groups 2, 3, or 4 were classified as high responders (HRs) (Figure 3B). The differences between these groups were statistically significant (*p* < 0.001): mean of HI titers: 179 ± 18 (HRs) vs. 50 ± 6 (LRs); mean of MN titers: 308 ± 28 (HRs) vs. 94 ± 11 (LRs).

**Figure 4:**
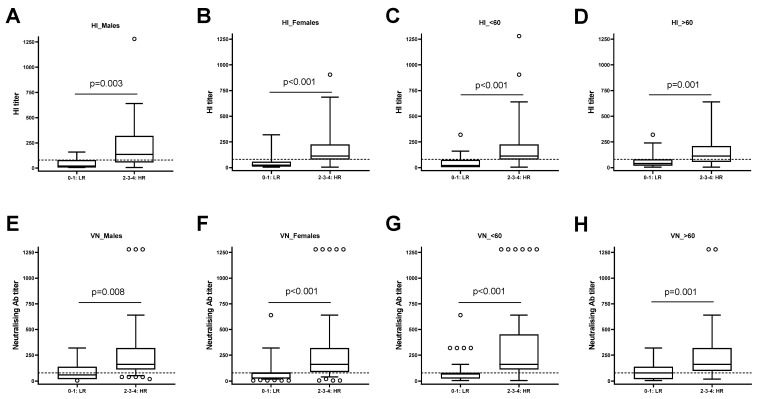
Stratification of subjects as low responders (LRs) and high responders (HRs) based on sex and age. The study population was stratified according to sex, male ((**A**) HI and (**E**) MN) and female ((**B**) HI and (**F**) MN) and to age, <60 ((**C**) HI and (**G**) MN) and ≥60 ((**D**) HI and (**H**) MN) years old. Dashed lines are means of medians of the four antigens and circles are outliers.The differences between LRs and HRs are significant in all analyzed subgroups (*p* < 0.01).

The same analysis was applied to the study population stratified by sex or age. We found that the differences between the HI titers of HRs and LRs were significant in males (*p* = 0.003) (Figure 4A) and females (*p* < 0.001) (Figure 4B). The same results were observed when stratifying the population into individuals with an age below 60 (*p* < 0.001) (Figure 4C) and individuals with more than 60 years (*p* < 0.001, Figure 4D). These data suggest that the results were similar independent of sex and age.

### 3.4. The High Responders to Influenza Vaccine Compared to the Low Responders Show a Strong Capacity to Respond to the SARS-CoV-2 Vaccine

**Figure 5:**
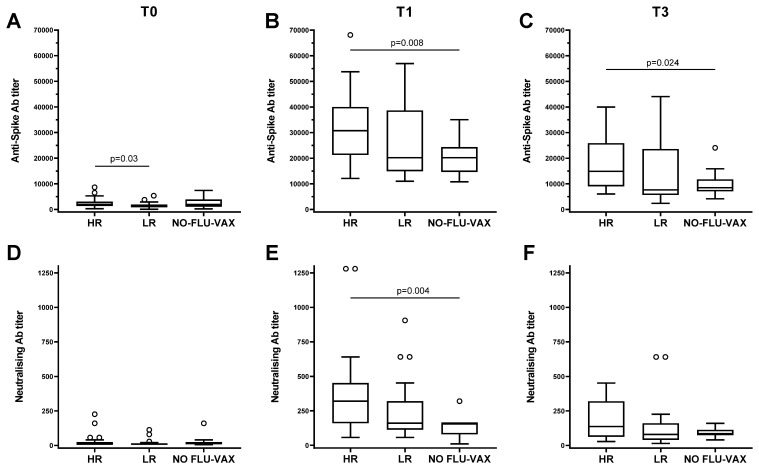
Effect of influenza vaccination on the response to the booster dose of SARS-CoV-2 vaccine in high (HRs) and low (LRs) responders. Anti-spike antibody titers (**A**–**C**) and neutralizing antibody titers (**D**–**F**) in HRs, LRs, and individuals who received only the anti-SARS-CoV-2 vaccine (NO-FLU-VAX) are shown before (T0) and at one month (T1) and three months post-SARS-CoV-2 vaccination. Circles are outliers. Statistically significant differences between groups are shown with their relative *p*-values.

To evaluate if the capacity to respond to the flu vaccine had any effect on the response to the booster dose of the SARS-CoV-2 vaccine, we compared the responses to the S protein of the HRs or LRs to the influenza vaccine and the ones who received only the SARS-CoV-2 vaccine either after one or three months post-vaccine injection. We found that the HRs have a strong capacity to respond to the SARS-CoV-2 vaccine after one month, whereas the LRs do not show any significant differences compared to the individuals who received only one vaccine (mean anti-spike antibody titer: 31,083 ± 2372 (HRs) vs. 20,823 ± 1804 (NO-FLU-VAX), *p* = 0.008) (Figure 5B). Furthermore, the HRs to the influenza vaccine showed a significantly higher response (mean anti-spike antibody titer: 16,600 ± 2050 (HRs) vs. 9745 ± 1212 (NO-FLU-VAX), *p* = 0.024) also after three months from the vaccine injection (Figure 5C), suggesting that the duration of the immune responses to the SARS-CoV-2 vaccine was longer when people received the influenza vaccine. The same results were found for the in vitro neutralization assay using the SARS-CoV-2 virus (mean neutralizing antibody titer after 4 weeks: 346 ± 57 vs. 158 ± 23, respectively, *p* = 0.004) (Figure 5E), whereas, although no significant differences, were observed the neutralization capacity was still higher in the HR individuals after three months (182 ± 31 (HRs) vs. 99 ± 11 (NO-FLU-VAX), *p* = 0.07). Of note, the baseline titer of anti-spike antibodies was not significantly different at T0 between the group of HRs to the influenza vaccine and the group who received only SARS-CoV-2 vaccine (2508 ± 340 vs. 2631 ± 664, *p* = 0.67) (Figure 5A), but a significant difference was observed between the baseline of HRs and of LRs to influenza vaccination (2508 ± 340 vs. 1638 ± 240, *p* = 0.03). These results further indicate that both extrinsic factors, such as the flu vaccine, and intrinsic factors related to the capacity to mount a good response to the vaccine are important for response to vaccination.

### 3.5. Analysis of the Effects of the Flu-Specific Antibody Response on Anti-Spike Antibody Levels in Double-Vaccinated Individuals

**Figure 6:**
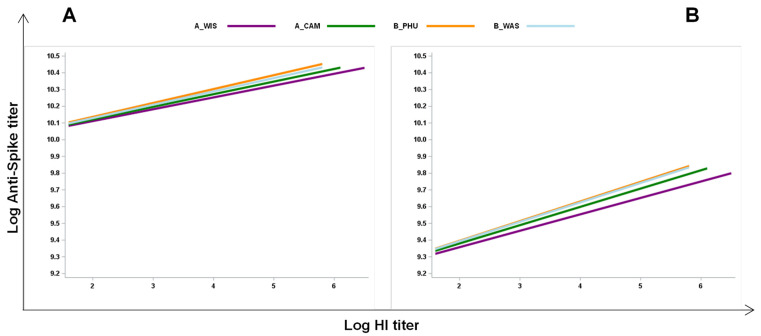
Positive association between the influenza-specific antibody response and anti-spike antibody levels in double-vaccinated participants. A linear regression model was applied considering values relative to samples collected after one (**A**) and three months (**B**) post-SARS-CoV-2 vaccination in participants who received both the quadrivalent influenza vaccine and the anti-SARS-CoV-2 vaccine. Each line represents the levels of anti-spike antibodies for the four different flu antigens. Statistical analysis reveals significant effects of flu-antigen on anti-spike antibodies at both time points T1 and T3 (1 and 3 months post-vaccination, respectively).

Next, to evaluate whether the extension of the response to the influenza vaccination could affect the capacity to respond to the SARS-CoV-2 vaccine by comparing the vaccine responses in the same subjects, we analyzed the associations of the responses in individuals who received two vaccines. A linear regression model was applied considering values relative to samples collected after one (Figure 6A) and three months (Figure 6B) post-SARS-CoV-2 vaccination. We found that there was a positive association between anti-S antibody titers and each specific flu antigen, with A/Wis: β = 0.071, *p* = 0.004; A/Cam: β = 0.075, *p* = 0.010; B/Phu: β = 0.083, *p* = 0.003; and B/Was: β = 0.080, *p* = 0.007, one month after vaccination. Furthermore, there was a positive association between anti-S and each flu-specific antigen, with A/Wis: β = 0.098, *p* = 0.023; A/Cam: β = 0.109, *p* = 0.035; B/Phu: β = 0.11, *p* = 0.003; B/Was: β = 0.116, *p* = 0.023 after three months following SARS-CoV-2 vaccination. These findings further suggest that influenza vaccination has a positive effect on the capacity to mount a good immune response to an unrelated vaccine.

## 4. Discussion

In this study, we found that the vaccination with the QIV improves the immune responses to the booster dose of the anti-SARS-CoV-2 vaccine in a cohort of healthy individuals. Furthermore, we observed that the intrinsic ability to respond to influenza vaccination correlates to the capacity to mount a strong antibody response to the anti-SARS-CoV-2 vaccination. In addition, the virus neutralization assay with an authentic SARS-CoV-2 virus showed that the anti-S antibodies are functional and confer protection against infection. These data are in accordance with several recent observational studies, which suggest a link between influenza vaccination and decreased COVID-19 incidence and severity [6,7,8]. The possibility of bias in epidemiological observations should be taken into account due to the inherent possibility of interfering factors that can cause over- or underestimation of outcomes. However, as a support of epidemiological observations, immunological evidence showed that the quadrivalent inactivated influenza vaccination is able to induce trained immunity by inducing a transcriptional and functional reprogramming of innate immune cells [20]. Indeed, it has been suggested that trained immunity might be an important mechanism underlying the protective heterologous effects of vaccines [21]. BCG is the most studied vaccine that induces trained immunity, and its putative protective effects against COVID-19 severity were studied in several clinical trials before the vaccines against SARS-CoV-2 were released [22,23]. Here, we found that influenza vaccination is able to potentiate SARS-CoV-2 vaccine efficacy, which has an impact on the protection from the disease in accordance with epidemiological and immunological studies [24,25,26]. On the other hand, whether influenza vaccination induces trained immunity in our cohort is currently under investigation. Considering the interval time between the two vaccinations, any significant time gap, which could be a factor in the levels of the response, was not found. However, we cannot exclude that an interval gap between the vaccines could be preferential; to assess this, more dedicated studies are needed. The responses to vaccinations are highly variable in the population, with some individuals who respond promptly and at high levels of antibodies and others who do not develop a protective response, and this is a common feature of different vaccines [27,28,29,30,31]. According to this, the participants included in this study responded with a significant increase in HI titers against all four antigens included in the vaccines, with high variability among individuals, as expected [10,32]. Of note, participants in this study responded better to the type A influenza antigens as compared to the antigens belonging to influenza virus type B. This could probably be related to the different immunogenicity of the antigens or to the variability in the laboratory analyses [33]. Furthermore, a high number of participants were already seroprotected at the baseline against the four antigens included in the vaccine. Differences between the percentage of protection at baseline were observed for the different viral strains, and a lower percentage of baseline protection was found for the A/Cambodia/H3N2 strain (20%). The high baseline titers are the results of previous exposures to the influenza virus or to earlier vaccinations. It has been widely reported that the baseline titers have an effect on the responses to the flu vaccine, with controversial results [14,34,35,36,37,38,39]. Here, we found that the baseline titer correlates with the response. Due to the different baseline titers among individuals, the identification of responders and non-responders following influenza vaccination is complex in both young and elderly populations [40,41]. Therefore, here, the population was stratified into five groups according to the capacity of the individuals to respond to the vaccination with an HI titer below or above the median of the HI of each viral antigen. Participants were classified as LRs and HRs. High responders to the flu vaccine have a stronger capacity to respond to the SARS-CoV-2 vaccine one and three months after vaccination, as compared to LRs, which do not show any significant differences if compared to the individuals who received only one vaccine. In addition, a positive association between influenza-specific antibody response and anti-S antibody levels was found in double-vaccinated individuals, further suggesting that influenza vaccination has a positive effect on the response to unrelated antigens. On the other hand, these findings are in accordance with the important role played by the intrinsic factors of the host in the responses to vaccinations and highlight the importance of analyzing the immune signatures measured prior to vaccination in order to identify potential biomarkers able to predict the vaccine immune responses [36]. Identifying the immune signature of individuals with high or low responsiveness to immunization holds the potential for optimizing existing vaccination strategies.

The limitations of this study are as follows: We are aware that the cohort is not large (74 eligible individuals) and there are two females per male ratio who received two vaccines; however, about the same proportion of two females per male ratio are present in the participants who received only SARS-CoV-2 vaccine. In addition, we would like to underline that participants were recruited at the time of a very high circulation rate of the SARS-CoV-2 virus in Italy, and at the beginning of this study, we enrolled more participants (113). About five blood samples for each participant were collected during this study, and at all these time points, we checked the levels of the anti-spike antibodies and the anti-nucleoprotein antibody levels to be aware of the infections occurred even if the participants were asymptomatic. By performing these controls, we excluded 26 participants who became infected during this study from the analysis and another 13 for other excluding factors.

In conclusion, this study reveals the association between influenza vaccination and the immune responses to the booster dose of the anti-SARS-CoV-2 vaccine and identifies individuals with high or low responsive capacity to the influenza vaccine, who exhibit different capabilities to face heterologous vaccine responses and potentially also infections and diseases. This could potentially identify individuals at risk and guide the development of targeted and personalized vaccination strategies.

## Figures and Tables

**Table 1:** Sociodemographic characteristics of the study population.

	Two Vaccines (n = 54, 73%)		One Vaccine (n = 20, 27%)		Total (n = 74)	
Gender	n.	%	n.	%	n.	%
Male	18	33	6	30	24	32
Female	36	67	14	70	50	68
Age						
Median	57		51		54	
IQR; range	(49–60); (35–70)		(49–56); (31–72)		(49–60); (31–72)	
Education						
Higher	41	76	12	60	53	72
Middle or lower	13	24	8	40	21	28
Frailty						
Very fit	8	15	7	35	15	20
Fit	39	72	12	60	51	69
Managing well	7	13	1	5	8	11
Body mass index						
Underweight	1	2	0	0	1	1
Healthy weight	30	55	12	60	42	57
Overweight	20	37	6	30	26	35
Obesity	3	6	2	10	5	7
Smoking						
Yes	7	13	5	25	12	16
No	47	87	15	75	62	84
Medical conditions						
Yes	23	43	11	55	34	46
No	31	57	9	45	40	54

## Data Availability

The data presented in this study are available upon request from the corresponding author. Due to the ongoing nature of this study, the data are not publicly available at this time.
